# Laparoendoscopic Single-Site Inguinal Herniorrhaphy: Experience of a Single Institute

**DOI:** 10.3390/jcm12051786

**Published:** 2023-02-23

**Authors:** Wei-Quen Tee, Yen-Ting Wu, Hung-Jen Wang, Yao-Chi Chuang, Wei-Chia Lee, Chia-Hung Tsai, Long-Yuan Lee, Chien-Hsu Chen

**Affiliations:** 1 Department of Urology, Chang Gung Memorial Hospital, Kaohsiung Medical Center, Chang Gung University, College of Medicine, Kaohsiung 83301, Taiwan; 2 Department of Leisure and Sport Management, Cheng Shiu University, Kaohsiung 83347, Taiwan

**Keywords:** inguinal hernia, laparoendoscopic single-site surgery (LESS), total extraperitoneal approach (TEP)

## Abstract

**Background**: Minimally invasive techniques for inguinal herniorrhaphy have focused on developing the laparoendoscopic single-site (LESS) procedure to improve cosmesis. Outcomes of total extraperitoneal (TEP) herniorrhaphy vary considerably because of being performed by different surgeons. We aimed to evaluate the perioperative characteristics and outcomes of patients undergoing the LESS-TEP approach for inguinal herniorrhaphy and to determine its overall safety and effectiveness. **Methods**: Data of 233 patients who underwent 288 laparoendoscopic single-site total extraperitoneal approach (LESS-TEP) herniorrhaphies at Kaohsiung Chang Gung Memorial Hospital between January 2014 and July 2021 were reviewed retrospectively. We reviewed the experiences and results of LESS-TEP herniorrhaphy performed by a single surgeon (CHC) using homemade glove access and standard laparoscopic instruments with a 50 cm long 30° telescope. **Results**: Among 233 patients, 178 patients had unilateral hernias and 55 patients had bilateral hernias. About 32% (n = 57) of patients in the unilateral group and 29% (n = 16) of patients in the bilateral group were obese (body mass index ≥ 25). The mean operative time was 66 min for the unilateral group and 100 min for the bilateral group. Postoperative complications occurred in 27 (11%) cases, which were minor morbidities except for one mesh infection. Three (1.2%) cases were converted to open surgery. Comparison of the variables between obese and non-obese patients found no significant differences in operative times or postoperative complications. **Conclusion**: LESS-TEP herniorrhaphy is a safe and feasible operation with excellent cosmetic results and a low rate of complication, even in obese patients. Further large-scale prospective controlled studies and long-term analyses are needed to confirm these results.

## 1. Introduction

Inguinal herniorrhaphy is one of the most frequently performed surgeries worldwide. Compared to conventional open repair surgery, laparoscopic inguinal herniorrhaphy has demonstrated promising outcomes, including alleviating postoperative pain, allowing an earlier return to normal activities with a shorter length of hospital stay, a better cosmetic result, and improved quality of life in the postoperative period [1,2]. However, it has a longer learning curve and higher costs [2].

A laparoscopic procedure generally has two approaches: transabdominal preperitoneal (TAPP) or total extraperitoneal (TEP). The TEP approach has a steep learning curve due to the cramped and unfamiliar visual field of the preperitoneal space. However, at the same time, the approach reduces unnecessary exposure of the bowel and the risk of serious visceral injury compared to TAPP [3].

Minimally invasive techniques have focused on developing the laparoendoscopic single-site (LESS) procedure to improve cosmetic outcomes. This single-incision technique is a less invasive alternative compared to conventional laparoscopic surgery, requiring only one incision over the umbilical fold. Each incision carries some risk of morbidities, such as bleeding and iatrogenic injury to the internal abdominal organ and vessels during the operation. Therefore, a greater number of incisions is associated with an increased risk of port-related morbidities and poorer cosmetic results [4,5]. Cosmesis is an important issue for many patients. A published survey of 750 patients highlighted that patients desire better cosmetic outcomes [6].

Previous studies regarding the outcomes of LESS-TEP vary considerably because different surgeons performed the procedures. In this study, the experiences and results of LESS-TEP performed in a single hospital by a single surgeon (CHC) have been reviewed.

The purpose of thisstudy was to evaluate the perioperative characteristics and outcomes of patients undergoing the extraperitoneal approach (LESS-TEP) for herniorrhaphy and to determine the overall safety and effectiveness of the procedure.

## 2. Patients and Methods

Data of patients who underwent a laparoendoscopic single-site total extraperitoneal approach (LESS-TEP) herniorrhaphy in Kaohsiung Chang Gung Memorial Hospital (KCGMH) between January 2014 and July 2021 were reviewed retrospectively. All procedures were performed by a single surgeon, Dr. C. H. Chen. The patients’ demographic data, body mass index (BMI), underlying history, history of intra-abdominal operations, American Society of Anesthesiologists (ASA) grade of physical status, hernia size, intraoperative data, and postoperative data were collected retrospectively. The study was approved by the Institutional Review Board of the KCGMH (No.: 202200718B0). Because of the retrospective nature of the study, signed informed consent of the patients was waived.

**Figure 1:**
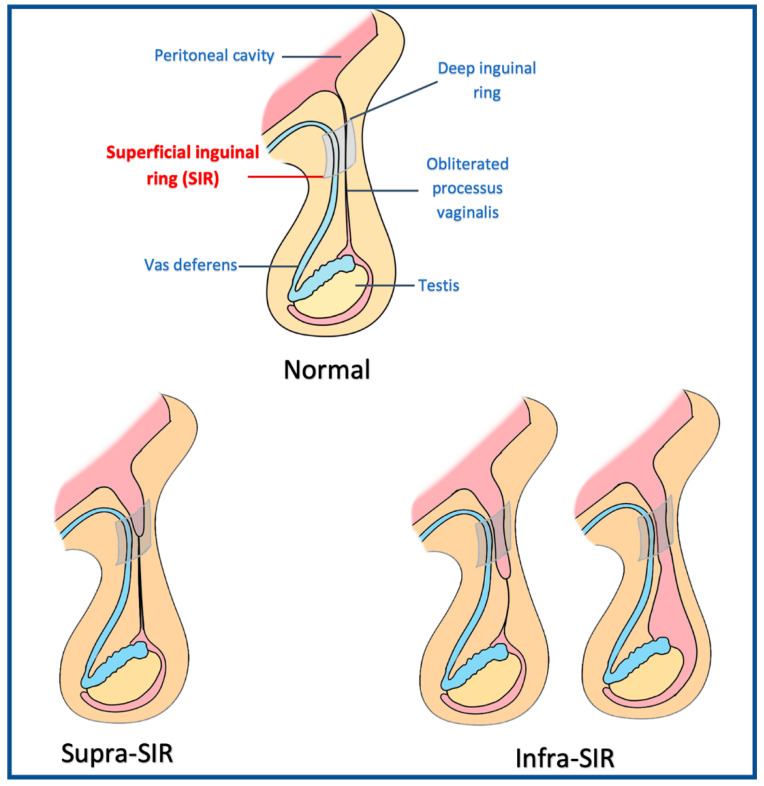
The size of the inguinal sac. The superficial inguinal ring (SIR) was defined as a boundary. The inguinal sac beyond the SIR or into the scrotum was the infra-SIR type, while the hernia sac above the SIR was the supra-SIR type.

Hernia size was evaluated in the outpatient department. When evaluating the sizes of the inguinal hernias, patients were requested to stand up and perform the Valsalva maneuver for at least 10 min. The superficial inguinal ring (SIR) was defined as a boundary. The inguinal sac beyond the SIR or into the scrotum was the infra-SIR type, while the hernial sac above the SIR was the supra-SIR type (Figure 1). The pantaloon hernia type referred to a coincidence of indirect and direct hernia over the ipsilateral groin.

The numerical pain rating scale was used to evaluate the pain scale on postoperative day 1. Pain killers with Non-Steroidal Anti-Inflammatory Drugs (NSAIDs) or acetaminophen were administered to all patients. Additional analgesics were defined as intravenous injections of parecoxib or intramuscular injections of morphine. All patients were required to return to the outpatient department within 7 days of discharge. The wound condition was evaluated during the outpatient department visit. If there was no morbidity, treatment was terminated. However, patients with complications had to revisit the outpatient department until the morbidity was resolved. Patients were also requested to revisit the outpatient department if hernia recurrence was suspected.

### 2.1. Surgical Technique

**Figure 2:**
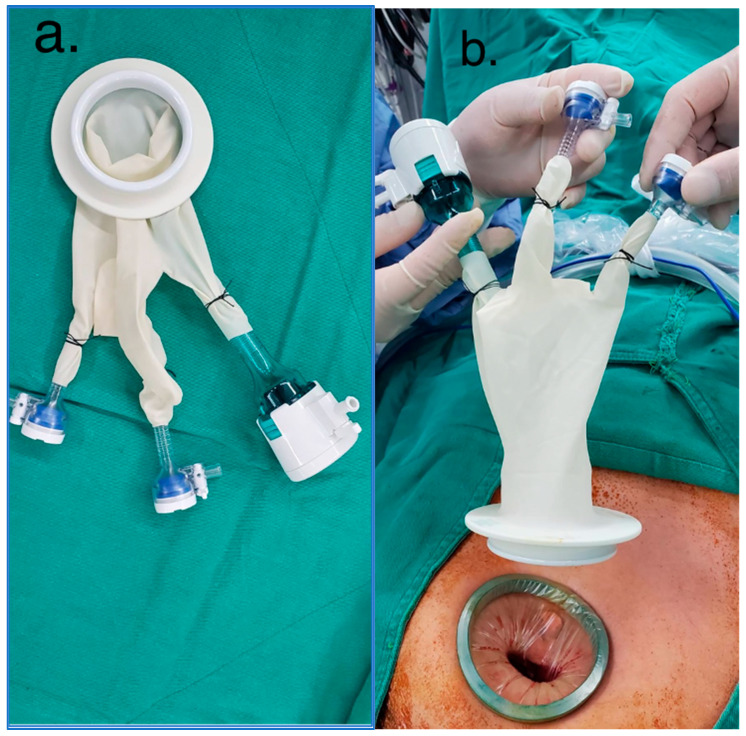
The homemade glove access and the wound retractor. (**a**) The homemade glove access was obtained using an 11 mm trocar and two 5 mm trocars; (**b**) The homemade glove access and the wound retractor (LAGIS^®^ WR-60ES) (before being attached).

The patient was placed in a supine position under general anesthesia. A 1.5–2 cm incision was made at the infra-umbilical edge as a quadrant circle line. The incision was carried down to the anterior sheath of the abdominal rectus. A slight space was created between the transversalis fascia and peritoneum. A balloon dilator was used to create a preperitoneal space. Then, a wound retractor (LAGIS^®^ WR-60ES) was placed and homemade powder-free glove access (Figure 2) using an 11 mm trocar and two 5 mm trocars were attached to the retractor. The preperitoneal space was insufflated to 10 mmHg. A 50 cm long Hopkins Forward-Oblique Telescope 30° was inserted and manipulated using laparoscopic dissectors.

**Figure 3:**
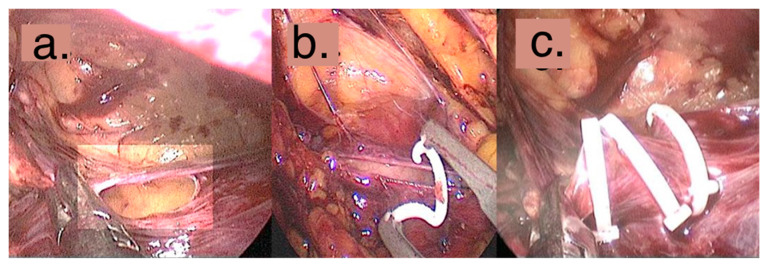
The peritoneal tear was repaired during the operation. (**a**) A 2 cm peritoneal tear; (**b**,**c**) Hem-o-lok was used to close the tear.

**Figure 4:**
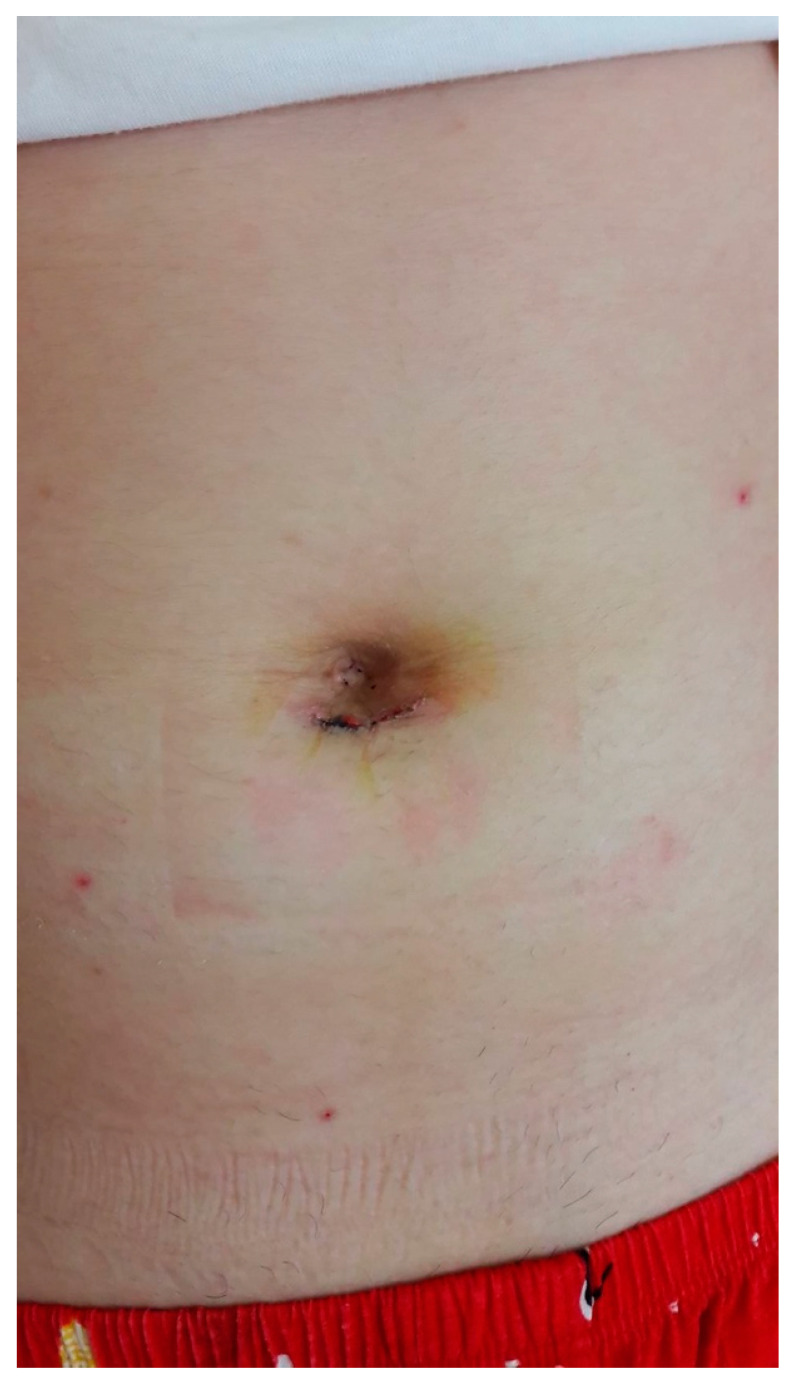
The wound condition during outpatient department follow-up.

The preperitoneal space was cleared out through the internal ring to identify landmarks such as the inferior epigastric vessel, pubic bone, and internal inguinal ring. After completing the preperitoneal dissection, sac isolation was performed. In the case of a large hernial sac or adhesion, the hernial sac was divided just beyond the internal inguinal ring, and then transected after the peritoneum was closed using the Weck Hem-o-lock polymer ligation system (Hem-o-lok, Teleflex, Wayne, PA, USA). If peritoneal tears were noted, they were closed using the Hem-o-lok (Figure 3) or sutures. After parietalization of the spermatic cord, mesh positioning was performed. A 15 × 10 cm parietex hydrophilic anatomical polyester mesh (Medtronic, Minneapolis, MN, USA) or 3 × 6 inch monofilament polypropylene mesh (Davol, Bard, Warwick, RI, USA) was placed without wrinkles. The choice of mesh was dependent on the patient’s economic status (the anatomical mesh costs around USD 520 while polypropylene mesh would be reimbursed by the Taiwanese National Health Insurance). We used an Absorbatack fixation device (Covidien, Medtronics, Minneapolis, MN, USA) to fix the monofilament polypropylene mesh. However, the parietex hydrophilic anatomical mesh was designed to encircle the gonadal vessels and vas deferens, which combined 2D and 3D weave to reduce mesh mobilization. Therefore, no fixation device was used to fix the parietex hydrophilic anatomical mesh. The space was deflated while monitoring the mesh to ensure it was in place. Then the homemade glove access and retractor were removed and the fascia was sutured layer by layer. The single skin incision was closed with subcuticular sutures (Figure 4).

### 2.2. Statistical Analysis

Statistical analysis was performed using IBM SPSS statistics Base 22.0 software (IBM Corp. Released 2013. IBM SPSS Statistics for Windows, Version 22.0 Armonk, NT, USA: IBM Corp). For the analysis of clinical characteristics and variables between obese and non-obese groups, we used an independent *t*-test for continuous variables and Chi-square or Fisher’s exact test for categorical variables. *p* < 0.05 was considered statistically significant.

## 3. Results

### 3.1. Patients’ Demographic and Clinical Data

A total of 288 LESS-TEP herniorrhaphies were performed on 233 patients (unilateral: 178 patients, bilateral: 55 patients). The patients’ demographic data and hernia characteristics are summarized in Table 1. Most patients were male with a mean age of 57 ± 14 years in the unilateral group and 64 ± 10 years in the bilateral group. About 32% (n = 57) of the patients in the unilateral group were obese (BMI ≥ 25), while 29% (n = 16) of the patients in the bilateral group were obese [7]. Most patients in both groups (unilateral: 75.3%; bilateral: 78.2%) had an ASA physical status of grade II. About 16% of patients had a history of abdominal surgery in the unilateral group and about 18% in the bilateral group. The history of abdominal surgeries included appendectomy, cholecystectomy, colectomy, and others. Twenty-three percent (n = 41) of patients in the unilateral group had an infra-SIR type hernia while 27% (n = 30) in the bilateral group had one.

### 3.2. Perioperative Data

The perioperative data were shown in Table 2. Mean operative time was 66 ± 26 min in the unilateral group and 100 ± 39 min in the bilateral group. The most common hernia type was indirect (75.8%) in the unilateral group while in the bilateral group it was the direct type (54.5%). Prior herniorrhaphy cases occurred in less than 5% of both groups. Incarcerated or femoral hernias were not found. Peritoneal tears were 19.6% in the unilateral group and 14.5% in the bilateral group. Any size of peritoneal perforation found during the surgery was marked as a peritoneal tear. The blood loss in this procedure was minimal and no patient needed blood transfusion. A total of three cases were converted to open surgery due to severe adhesion.

### 3.3. Postoperative Outcomes and Complications

Postoperative outcomes and complications are listed in Table 3. The mean hospital stay after surgery was 1 day in both groups. Two patients underwent outpatient surgeries in the unilateral group. The mean numerical pain rating scales on postoperative day 1 in unilateral and bilateral group were 1.6 and 1.9, respectively. A total of 16.85% in the unilateral group and 12.7% in the bilateral group of patients needed an additional analgesic agent. Prolonged spermatic cord pain was defined as the need for oral painkillers one week after surgery. Prolonged spermatic cord pain was 6.18% in the unilateral group and 10.9% in the bilateral group. Among the seven cases that experienced inguinal seroma, two required fine-needle percutaneous aspiration while others resolved spontaneously. The median follow-up period was 1.14 weeks (range, 1~326). One patient experienced a delayed mesh abscess 3 years postoperatively. He was treated successfully with mesh removal and debridement. In this study, only one patient experienced recurrence at the surgical side one year after surgery and he received open herniorrhaphy for recurrence. However, five patients experienced recurrence over another side (non-surgical side) and all of them received open repair in consideration of adhesion due to prior laparoscopic surgery. Currently, we routinely check the contralateral side when performing unilateral LESS-TEP. If a contralateral hernia is noted during surgery, we perform bilateral repair to avoid reoperation in the future.

In the unilateral group, the perioperative and postoperative data between obese patients (BMI ≥ 25) and non-obese patients (BMI < 23) are listed in Table 4. There were 57 patients in the obesity group and 60 patients in the non-obesity group. No significant differences were found between the two groups in the parameters of hernia size, operative time, pain rating scale, and complications.

## 4. Discussion

Controversy still exists about the best surgical repair for inguinal hernias. Therefore, we undertook this study because we considered it worthwhile from the perspective of LESS-TEP safety to clarify the outcomes of LESS-TEP. We generally favored LESS-TEP because we were able to manipulate the hernial sac without going through the intra-peritoneal cavity, which lowered the risk of complications such as visceral injury, intestinal obstruction, and port-site hernia, as previously described [3,8,9]. In the recent literature, long-term outcomes of chronic groin pain, hernia recurrence, and quality of life were comparable between TEP and TAPP [10,11]. Another systematic review and meta-analysis showed comparable surgical efficacy and morbidity between LESS-TEP and the conventional multiple-port TEP, except for cosmesis [12]. Therefore, the present study focused on LESS-TEP and reviewed the outcomes of surgical cases that were performed at our institution.

In this study, the total complication rate was 11%, which was compatible with those of previous studies [13,14,15]. We defined prolonged spermatic cord pain as spermatic cord induration and continued pain requiring painkillers for relief over one week after surgery. In most patients, painkillers (such as NSAIDs or acetaminophen) were prescribed for 3 days. This pain may be the result of transient inflammation due to intraoperative hernia sac dissection. Roland et al. reported that reduction of the hernia surface area may cause chronic pain [16]. However, all patients with prolonged spermatic cord pain experienced subsided pain 2–3-weeks after postoperative follow-up.

The incidence of seroma formation after herniorrhaphy varies from 0.5% to 12.2% [3]. The risk factors for seroma formation are coagulopathy, congestive liver disease, and cardiac insufficiency [17]. Patients can develop fluid accumulation at the space where the hernia sac used to be and the fluid reabsorbs spontaneously with time in most cases. Therefore, most seromas resolve spontaneously in 6–8 weeks [3]. In the present study, seven seroma cases were noted and two of them needed percutaneous fine-needle aspiration. The factors for reducing seroma formation included a complete reduction of the hernial sac without being transected. A recent prospective randomized controlled study showed that a left-in-situ transected hernial sac might increase exudation, resulting in seroma formation [18].

In this study, a total of three cases were converted to open surgeries with a conversion rate of 1.28%, comparable to the rates reported for previous studies (0.48–1.8%) [13]. Two patients had adhesive preperitoneal space due to the previous herniorrhaphy; the other had bulky omentum incarceration. It is known that the abdominal cavity is more likely to develop adhesion when the patient has had previous abdominal operations. We created a preperitoneal cavity to manipulate the hernial sac in this limited space. Therefore, the difficulty of the surgery and the operative time would increase if the patient had had a previous lower abdominal operation [19]. The data between obese and non-obese patients in the unilateral group were also comparable. Although we supposed that the operation time would be relatively higher in the obese group, the results showed no significant differences between the two groups. In a previous study, high BMI correlated with prolonged operative times only during the surgeon’s learning period. Upon reaching the experienced level, the surgeon appeared to handle the challenge easily [20].

Variable access ports are placed at single-incision wounds, such as multi-instrument access or single-access ports. The access port may restrict the operating space of the TEP procedure [21]. Previous studies showed that homemade glove ports were safe and feasible [22,23]. We favored the use of a homemade powder-free glove port, which was easier to make and cost-effective. Due to the texture and elasticity, the homemade glove port provided greater angulation for manipulating the hernial sac. It could also be insufflated to make the tract clearer and larger. There was no significant gas leakage or glove rupture noted during the surgery under 10 mmHg pressure. In addition, the crowded laparoscopic instruments might lead to crashing, and therefore a 50 cm long Hopkins Forward-Oblique Telescope 30° was introduced that increased the working space for the surgeon and camera assistant, thereby reducing the crashing issue.

There are many different groin hernia classifications in the literature. However, most of them are complex and difficult to remember. Frequently used classifications such as Gilbert’s [24] and Nyhus’ [25] classifications were based on findings during an open (anterior) approach. The European hernia society (EHS) provided a general and systemic use of hernia classification [26]. However, the EHS classification system did not include the size or descent of the hernia sac. The hernia sac with scrotal extension is a major challenge during surgery, and it is important to determine this feature before surgery. Thus, we used the superficial inguinal ring as a boundary to evaluate the descending level of the hernia sac.

### Limitations

This study has several limitations, including first, that the sample size for this retrospective study was relatively low and limited to one center. Second, all the procedures were performed by a single experienced surgeon, limiting the generalizability of the results to other surgeons. However, the same surgeon performed all the operations, which reduces possible technique bias. Third, due to the short follow-up period, we may underestimate the recurrence rate and some delayed complications. Lastly, we did not analyze the factors that cause complications and affect operative time.

## 5. Conclusions

In conclusion, LESS-TEP herniorrhaphy is a safe and feasible operation with acceptable outcomes. Consequently, it may be a good option for those who care about wound cosmesis. Further large-scale prospective controlled studies and long-term analysis are still needed to further examine related chronic pain, recurrence rates, and effects on quality of life.

## Figures and Tables

**Table 1:** Patients’ demographics and hernia characteristics.

	Unilateral	Bilateral
Number of patients	178	55
Male	166	55
Female	12	0
Number of herniorrhaphies	178	110
Age (years), mean + SD	57 ± 14	64 ± 10
Body mass index (%)		
<25 (kg/m^2^)	121 (68%)	39 (71%)
≥25 (kg/m^2^)	57 (32%)	16 (29%)
ASA * (%)		
I	26 (14.6%)	2 (3.6%)
II	134 (75.3%)	43 (78.2%)
III	18 (10.1%)	10 (18.2%)
History of abdominal operation (%)	29 (16.3%)	10 (18.2%)
Hernia size (%)		
Supra-SIR	137 (77%)	80 (72%)
Infra-SIR	41 (23%)	30 (27%)

* ASA—American Society of Anesthesiologists grade of physical status. SIR—superficial inguinal ring.

**Table 2:** Perioperative data.

	Unilateral	Bilateral
Operative time (mins)	66 ± 26	100 ± 39
Hernia type (%)		
Direct	34 (13.5%)	60 (54.5%)
Indirect	135 (75.8%)	42 (38.2%)
pantaloon	5 (2.8%)	4 (3.6%)
prior herniorrhaphy with recurrence	4 (2.2%)	4 (3.6%)
Peritoneal tear (%)	35 (19.6%)	8 (14.5%)
Mesh (%)		
Parietex hydrophilic anatomical, polyester mesh (Medtronic, USA)	111 (62.3%)	10 (9.1%)
Lightweight monofilament polypropylene mesh (Davol, Bard, USA)	67 (37.6%)	100 (90.9%)
Conversion to open procedure (%)	1 (0.5%)	2 (1.18%)

**Table 3:** Postoperative outcomes and complications.

	Unilateral (n = 178)	Bilateral (n = 55)	*p*
Hospital stays after surgery (days), mean ± SD	1.2 ± 0.5	1.3 ± 0.5	0.215
Numerical pain rating scale, mean ± SD	1.6 ± 0.9	1.9 ± 1	0.138
Number of patients using additional analgesics *	30 (16.9%)	7 (12.7%)	0.243
Number of complications			
Seroma	5 (2.8 %)	2 (3.6%)	0.237
Prolonged Spermatic cord pain	11 (6.2%)	6 (10.9%)	0.243
Urinary retention	2 (1.1%)	0	0.999
Delayed abscess	0	1 (1.8%)	0.236

* Additional analgesics are defined as intravenous injection of parecoxib or intramuscular injection of morphine.

**Table 4:** Comparisons between obesity and non-obesity in the unilateral group.

	Obesity (BMI ≥ 25) (n = 57)	Non-Obesity (BMI < 23) (n = 60)	*p*
Hernia size (%)			0.558
Supra-SIR	41 (71.9%)	46 (76.7%)	
Infra-SIR	16 (28.1%)	14 (23.3%)	
Peritoneal tear (%)	9 (15.8%)	17 (28.3%)	0.103
Operative time (mins)	68.6 ± 23	66.5 ± 30.1	0.671
Hospitalization (days), mean ± SD	1.2 ± 0.5	1.2 ± 0.5	0.788
Numerical pain rating scale, mean ± SD	1.6 ± 0.9	1.8 ± 0.9	0.255
Number of complications			
Seroma	2 (3.5%)	1 (1.7%)	0.612
Prolonged spermatic cord pain	4 (7.0%)	2 (3.3%)	0.431
Urinary retention	1 (1.8%)	1 (1.7%)	0.999

## Data Availability

Data sharing not applicable.

## References

[1] Memon M.A., Cooper N.J., Memon B., Memon M.I., Abrams K.R. (2003). Meta-analysis of randomized clinical trials comparing open and laparoscopic inguinal hernia repair. Br. J. Surg..

[2] Ielpo B., Nunez-Alfonsel J., Duran H., Diaz E., Fabra I., Caruso R., Malavé L., Ferri V., Barzola E., Quijano Y. (2018). Cost-effectiveness of Randomized Study of Laparoscopic Versus Open Bilateral Inguinal Hernia Repair. Ann. Surg..

[3] HerniaSurge G. (2018). International guidelines for groin hernia management. Hernia.

[4] Ahmed I., Paraskeva P. (2011). A clinical review of single-incision laparoscopic surgery. Surgeon.

[5] Kommu S.S.R.A. (2009). Devices for laparoendoscopic single-site surgery in urology. Expert Rev. Med. Devices.

[6] Rao A., Kynaston J., MacDonald E.R., Ahmed I. (2010). Patient preferences for surgical techniques: Should we invest in new approaches?. Surg. Endosc..

[7] World Health Organization, Regional Office for the Western Pacific (2000). The Asia-Pacific Perspective: Redefining Obesity and Its Treatment.

[8] Bringman S., Blomqvist P. (2005). Intestinal obstruction after inguinal and femoral hernia repair: A study of 33,275 operations during 1992–2000 in Sweden. Hernia.

[9] McCormack K., Wake B.L., Fraser C., Vale L., Perez J., Grant A. (2005). Transabdominal pre-peritoneal (TAPP) versus totally extraperitoneal (TEP) laparoscopic techniques for inguinal hernia repair: A systematic review. Hernia.

[10] Bansal V.K., Misra M.C., Babu D., Victor J., Kumar S., Sagar R., Rajeshwari S., Krishna A., Rewari V. (2013). A prospective, randomized comparison of long-term outcomes: Chronic groin pain and quality of life following totally extraperitoneal (TEP) and transabdominal preperitoneal (TAPP) laparoscopic inguinal hernia repair. Surg. Endosc..

[11] Aiolfi A., Cavalli M., Del Ferraro S., Manfredini L., Lombardo F., Bonitta G., Bruni P.G., Panizzo V., Campanelli G., Bona D. (2021). Total extraperitoneal (TEP) versus laparoscopic transabdominal preperitoneal (TAPP) hernioplasty: Systematic review and trial sequential analysis of randomized controlled trials. Hernia.

[12] Lo C.W., Yang S.S., Tsai Y.C., Hsieh C.H., Chang S.J. (2016). Comparison of laparoendoscopic single-site versus conventional multiple-port laparoscopic herniorrhaphy: A systemic review and meta-analysis. Hernia.

[13] Lee Y.J., Kim J.H., Kim C.H., Lee G.R., Lee Y.S., Kim H.J. (2021). Single incision laparoscopic totally extraperitoneal hernioplasty: Lessons learned from 1231 procedures. Ann. Surg. Treat. Res..

[14] Suzuki Y., Wakasugi M., Mikamori M., Tamaoka K., Nakahara Y., Tei M., Furukawa K., Ohtsuka M., Masuzawa T., Akamatsu H. (2022). Long-term outcomes of single-incision versus multiport laparoscopic totally extra-peritoneal inguinal hernia repair: A single-institution experience of 186 consecutive cases. Surg. Today.

[15] Li C.-C., Tseng S.-I., Lee H.-Y., Chueh K.S., Tsai C.C., Chou Y.H., Huang C.N., Wu W.J. (2020). Retrospective comparison of open- versus single-incision laparoscopic extraperitoneal repair of inguinal hernia procedures: A single-institution experience. Urol. Sci..

[16] Kocijan R., Sandberg S., Chan Y.W., Hollinsky C. (2010). Anatomical changes after inguinal hernia treatment: A reason for chronic pain and recurrent hernia?. Surg. Endosc..

[17] Bhangu A., Singh P., Pinkney T., Blazeby J.M. (2015). A detailed analysis of outcome reporting from randomised controlled trials and meta-analyses of inguinal hernia repair. Hernia.

[18] Ruze R., Yan Z., Wu Q., Zhan H., Zhang G. (2019). Correlation between laparoscopic transection of an indirect inguinal hernial sac and postoperative seroma formation: A prospective randomized controlled study. Surg. Endosc..

[19] Dulucq J.L., Wintringer P., Mahajna A. (2006). Totally extraperitoneal (TEP) hernia repair after radical prostatectomy or previous lower abdominal surgery: Is it safe? A prospective study. Surg. Endosc..

[20] Kato J.M., Iuamoto L.R., Suguita F.Y., Essu F.F., Meyer A., Andraus W. (2017). Impact of Obesity and Surgical Skills in Laparoscopic Totally Extraperitoneal Hernioplasty. Arq. Bras. Cir. Dig..

[21] Kim J.H., Park S.M., Kim J.J., Lee Y.S. (2011). Initial experience of single port laparoscopic totally extraperitoneal hernia repair: Nearly-scarless inguinal hernia repair. J. Korean Surg. Soc..

[22] Tai H.C., Lin C.D., Wu C.C., Tsai Y.C., Yang S.S. (2010). Homemade transumbilical port: An alternative access for laparoendoscopic single-site surgery (LESS). Surg. Endosc..

[23] Tai H.C., Ho C.H., Tsai Y.C. (2011). Laparoendoscopic single-site surgery: Adult hernia mesh repair with homemade single port. Surg. Laparosc. Endosc. Percutaneous Tech..

[24] Gilbert A.I. (1989). An anatomic and functional classification for the diagnosis and treatment of inguinal hernia. Am. J. Surg..

[25] Nyhus L.M., Klein M.S., Rogers F.B. (1991). Inguinal hernia. Curr. Probl. Surg..

[26] Miserez M., Alexandre J.H., Campanelli G., Corcione F., Cuccurullo D., Pascual M.H., Hoeferlin A., Kingsnorth A.N., Mandala V., Palot J.P. (2007). The European hernia society groin hernia classification: Simple and easy to remember. Hernia.

